# Recent progresses in molecular postharvest biology

**DOI:** 10.1186/s43897-022-00040-1

**Published:** 2022-08-10

**Authors:** Su-Sheng Gan

**Affiliations:** Plant Biology Section, School of Integrative Plant Science, Cornell University, Ithaca, NY 14853 USA

Postharvest biology of horticultural crops is a scientific discipline of the biological processes that occur in horticultural crops after harvest. It is the foundation for practical strategies and technology for prolonging the postharvest longevity of produce. The postharvest longevity includes storage, logistics and transportation, and shelf life. It is estimated that up to 70% of produce are lost after harvest. Understanding of postharvest biological processes will allow us to devise ways to control these processes for improving the produce quality (and quantity) and reducing the economic loss.

Molecular biology and related techniques have been increasingly used in almost all aspects of horticulture research including postharvest biological studies (Gan and Xue 2021), which has significantly advanced our understanding of molecular genetic regulatory mechanisms of the physiological and biochemical changes in horticultural crops after harvest. Significant advances have been made in this regard, and we are pleased to present a special collection named “**Molecular Postharvest Biology**” to reflect the advances.

The Molecular Postharvest Biology special collection consists of three review articles (Guo et al. 2021; Sun et al. 2021; Zhang et al. 2021) and five research articles (Hu et al. 2021; Wang et al. 2021, 2022a, 2022b; Zou et al. 2021) that spans leaves, flowers, fruits, and postharvest pathogens.

**Leaves** are not only the photosynthetic organ but also constitute the major consumable part of foliar vegetables. Senescence of leaves limits yield and reduces the nutritional value of vegetables, especially foliar vegetables. Because of the significance much effort has been made to decipher the mechanisms underlying leaf senescence. Among the special collection are four articles on leaf senescence (Guo et al. 2021; Hu et al. 2021; Wang et al. 2022a, 2022b). The review article “Leaf senescence: progression, regulation, and application” by Guo et al. (2021) summarizes recent advances and breakthroughs in leaf senescence research with an emphasis on the molecular (epi)genetic mechanisms underlying chlorophyll degradation, transcriptional, posttranscriptional, hormonal and environmental regulation of senescence-associated genes (*SAG*s) and related biological processes. It also discusses novel strategies and technologies for delaying senescence that have been derived from the molecular understanding of the senescence processes. Future directions are also presented. AtNAP is one of the most important transcription factors discussed in the Review. Two research articles in the collection report the most recent findings that AtNAP promotes leaf senescence by activating the gene of cytokinin oxidase 3 (that degrades senescence-inhibiting plant hormone cytokinins) (Hu et al. 2021) and genes involved in biosynthesis of the senescence-inducing hormone salicylic acid (Wang et al. 2022b), demonstrating the sophisticated regulatory mechanism of leaf senescence by the AtNAP transcription factor. The research article by Wang et al. (2022a) reveals a new function of the dehydratase-enolase-phosphatase complex 1 (DEP1) in apple leaf senescence. DEP1 was initially shown to be involved in the Yang cycle for ethylene biosynthesis.

**Flowers** are “food for the mind,’ and senescence of flowers limits vase life and reduces aesthetic appreciation. In this special collection there are one review article (Sun et al. 2021) and one research article (Zou et al. 2021) representing the frontiers in flower senescence research. Many flowers, especially cut flowers such as rose, undergo opening and senescence processes after harvest. The review article entitled “Molecular understanding of postharvest flower opening and senescence” critiques and synthesizes literature on recent findings and breakthroughs in molecular genetic regulation of flower opening and senescence, presents integrated models of both processes, and offers future research directions in these areas (Sun et al. 2021). The research article by Zou et al. reports the identification and functional characterization of a transcription factor gene named *RhNAP* in rose petal senescence and drought resistance, and unravels the underlying mechanism by which RhNAP activates a cytokine oxidase gene to degrade cytokinins (Zou et al. 2021). It should be noted that *RhNAP* is an orthologue of *AtNAP* discussed above, and both NAPs function by directing the expression of cytokine oxidase genes to facilitate senescence processes in rose petals and Arabidopsis leaves, respectively (Hu et al. 2021; Zou et al. 2021). By the way, the inclusion of the two NAP-related research articles in the special collection well explains Molecular Horticulture’s decision to “publish research articles involving model plants that reveal mechanisms/principles readily applicable to horticultural plants” (Gan and Xue 2021).

**Fruits**, like vegetables, are very important part of human diet. Fruit ripening is associated with remarkable physiological and biochemical changes (Wang and Seymour 2022; Zhu et al. 2022) that are believed to be driven by thousands of genes, including many transcription factor genes. Among the transcription factors is Dof (DNA-binding with one finger) domain proteins that exists in plants only. This special collection presents a research article on the role and regulatory mechanism of *Dof1* transcription factor in tomato fruit ripening (Wang et al. 2021). *Dof1* has a key role in fruit ripening because the fruit ripening processes are delayed and the expression of hundreds of genes is altered in tomato when the gene is knocked down (Wang et al. 2021). More importantly, the authors use chromatin immunoprecipitation sequencing and identify more than 300 Dof-directly-targeted genes including some well-known fruit ripening-related genes such as *ACS2* and *PG2A* (Wang et al. 2021).

**Postharvest pathogens** infecting fruits and vegetables (as well as some animal roughages) are predominantly fungi (and *Erwinia carotovora* subsp. *carotovora* is the only postharvest bacterial pathogen) that render food (and feed) unsafe and contribute to significant loss of produce. Molecular understanding of pathogens and pathogenesis will help control the postharvest diseases. The special collection proudly presents a review article entitled “Molecular basis of pathogenesis of postharvest pathogenic fungi and control strategy in fruits: progress and prospect” (Zhang et al. 2021). The review brings to our attention the current research advances in postharvest pathology at molecular level, including genes, proteins, small RNAs and metabolites that constitute the regulatory bases of pathogenicity and/or pathogenesis. The review also lists and examines various molecular approaches for the control of postharvest diseases. The future direction is also offered (Zhang et al. 2021).

In summary, the special collection on “Molecular Postharvest Biology” with mix of review and research articles represents fore frontiers in this field, and will be excellent references for researchers and a textbook for advanced trainees.
