# Correction: Recent progresses in molecular postharvest biology

**DOI:** 10.1186/s43897-022-00041-0

**Published:** 2022-08-31

**Authors:** Su-Sheng Gan

**Affiliations:** Plant Biology Section, School of Integrative Plant Science, Cornell University, Ithaca, NY 14853 USA

**Correction: Mol Hortic 2, 18 (2022)**

**https://doi.org/10.1186/s43897-022-00040-1**

After publication of this article (Gan 2022), it was brought to our attention that some DOIs in the references are incorrect, here below are the correct references:

- Gan S-S, Xue H-W. Horticulture in a molecular age. Mol Hortic. 2021;1:1. 10.1186/s43897-021-00007-8.
- Guo Y, Ren G, Zhang K, Li Z, Miao Y, Guo H. Leaf senescence: progression, regulation, and application. Mol Hortic. 2021;1:5. 10.1186/s43897-021-00006-9.
- Hu Y, Liu B, Ren H, Chen L, Watkins CB, Gan S-S. The leaf senescence-promoting transcription factor AtNAP activates its direct target gene *CYTOKININ OXIDASE 3* to facilitate senescence processes by degrading cytokinins. Mol Hortic. 2021;1:12. 10.1186/s43897-021-00017-6.
- Sun X, Qin M, Yu Q, Huang Z, Xiao Y, Li Y, Ma N, Gao J. Molecular understanding of postharvest flower opening and senescence. Mol Hortic. 2021;1:7. 10.1186/s43897-021-00015-8.
- Wang D, Seymour GB. Molecular and biochemical basis of softening in tomato. Mol Hortic. 2022;2:5. 10.1186/s43897-022-00026-z.
- Wang Y, Wang P, Wang W, Kong L, Tian S, Qin G. Genome-wide binding analysis of the tomato transcription factor SlDof1 reveals its regulatory impacts on fruit ripening. Mol Hortic. 2021;1:9. 10.1186/s43897-021-00011-y.
- Wang C-K, Li X-M, Dong F, Sun C-H, Lu W-L, Hu D-G. Yang cycle enzyme DEP1: its moonlighting functions in PSI and ROS production during leaf senescence. Mol Hortic. 2022a;2:10. 10.1186/s43897-022-00031-2.
- Wang Y, Liu B, Hu Y, Gan S-S. A positive feedback regulatory loop, SA-*AtNAP*-*SAG202*/*SARD1*-*ICS1*-SA, in SA biosynthesis involved in leaf senescence but not defense response. Mol Hortic. 2022b;2:15. 10.1186/s43897-022-00036-x.
- Zhang Z-Q, Chen T, Li B-Q, Qin G-Z, Tian S-P. Molecular basis of pathogenesis of postharvest pathogenic fungi and control strategy in fruits: progress and prospect. Mol Hortic. 2021;1:2. 10.1186/s43897-021-00004-x.
- Zhu F, Wen W, Cheng Y, Fernie AR. The metabolic changes that effect fruit quality during tomato fruit ripening. Mol Hortic. 2022;2:2. 10.1186/s43897-022-00024-1.
- Zou J, Lu P, Jiang L, Liu K, Zhang T, Chen J, Yao Y, Cui Y, Gao J, Zhang C. Regulation of rose petal dehydration tolerance and senescence by RhNAP transcription factor via the modulation of cytokinin catabolism. Mol Hortic. 2021;1:13. 10.1186/s43897-021-00016-7.
